# Late urinary toxicity after prostate intensity-modulated radiation therapy for patients with history of invasive interventions for prostate or bladder

**DOI:** 10.1007/s11604-026-01969-9

**Published:** 2026-03-14

**Authors:** Koichi Kato, Rihito Aizawa, Takashi Ogata, Takayuki Goto, Kimihiko Masui, Yuki Kita, Takayuki Sumiyoshi, Kei Mizuno, Takashi Kobayashi, Takashi Mizowaki

**Affiliations:** 1 Department of Radiation Oncology and Image-Applied Therapy, Graduate School of Medicine, Kyoto University, 53 Kawahara-cho, Shogoin, Sakyo-ku, Kyoto, 606-8507 Japan; 2 Department of Urology, Graduate School of Medicine, Kyoto University, 53 Kawahara-cho, Shogoin, Sakyo-ku, Kyoto, 606-8507 Japan

**Keywords:** Prostate cancer, Intensity-modulated radiation therapy, Genitourinary toxicity, Invasive interventions for prostate or bladder

## Abstract

**Purpose:**

The aim of this study was to evaluate late genitourinary (GU) toxicities on receiving intensity-modulated radiation therapy (IMRT) in patients with prostate cancer (PCa) who had a history of invasive interventions for the prostate or bladder.

**Materials and methods:**

Among patients who received IMRT for PCa at our institution between August 2000 and December 2022, clinical outcomes among those with a history of invasive interventions for the prostate or bladder were retrospectively analyzed. Cumulative incidence rates of late ≥ grade 2 and ≥ grade 3 GU and gastrointestinal toxicities, and rates of overall survival (OS) and biochemical failure-free survival (BFFS) were evaluated.

**Results:**

A total of 32 consecutive patients were analyzed, among whom 28 received conventional fractionated IMRT with a median dose of 74 Gy in 37 fractions, and 4 underwent moderately hypo-fractionated IMRT with a median dose of 54 Gy in 15 fractions. The median follow-up period was 77.3 months after IMRT. Cumulative incidence rates of ≥ grade 2 and ≥ grade 3 GU toxicities were 20.7 and 4.3% at 5 years, and 26.4 and 4.3% at 8 years, respectively. More than 10 years after IMRT, 18.8% of patients developed ≥ grade 2 GU toxicities. OS and BFFS rates were 89.5 and 76.0% at 5 years, and 89.5 and 60.7% at 8 years, respectively.

**Conclusion:**

Prostate IMRT for patients with a history of invasive interventions for the prostate or bladder was considered a safe and feasible treatment option, although the incidence of late GU toxicities was relatively high. Long-term follow-up with close attention to the detection of GU toxicities is recommended for such a population.

**Secondary abstract:**

Clinical outcomes of IMRT in 32 patients with prostate cancer and a history of invasive interventions for the prostate or bladder were retrospectively analyzed. Although the incidence of late genitourinary toxicities was relatively high, prostate IMRT was considered a safe and feasible treatment option for such a population.

## Introduction

Prostate cancer (PCa) is the second most common cancer in males [1], and the incidence rate has consistently increased [2]. Radiation therapy (RT) is one of the standard treatment modalities for its definitive treatment. Although favorable clinical outcomes following external radiation therapy (EBRT) and brachytherapy have been reported [3–6], RT-related late genitourinary (GU) toxicities have caused concern despite their relatively low incidence rates. RT-related late GU toxicities can be symptomatic and difficult to manage, consequently impairing the quality of life of patients [7–9].

Risk factors for late GU toxicities include a history of invasive interventions for the prostate or bladder, such as: trans-urethral resection of prostate (TURP), holmium laser nucleation of prostate (HoLEP), transurethral resection of bladder tumor (TURBT), high intensity focused ultrasound (HIFU), transurethral enucleation with bipolar (TUEB) and subcapsular prostatectomy for benign prostatic hyperplasia (BPH) [10–12]. The incidence of severe late GU toxicities generally increases with time after irradiation [7]. However, toxicity data from patients with a history of such invasive interventions is primarily limited to studies with a median follow-up period of less than five years. Therefore, data from a longer follow-up period are needed to evaluate safety for those patients [12, 13].

Thus, the purpose of this study was to evaluate long-term clinical outcomes on receiving prostate IMRT, with a focus on late GU toxicities, among patients with a history of invasive interventions for the prostate or bladder.

## Materials and methods

This study followed the tenets of the Declaration of Helsinki, with approval from our institutional ethical review board (approval number: R1048-3). Written informed consent was not necessary due to the retrospective nature of this study. Instead, an opt-out option was made available on our website, and those who opted out were excluded from analysis.

### Patient selection

This retrospective study included patients with a history of invasive interventions for the prostate or bladder before intensity-modulated radiation therapy (IMRT) for PCa. To identify them, we reviewed the medical records of 1609 consecutive patients treated with definitive IMRT for PCa at our institution between August 2000 and December 2022. The invasive interventions included TURP, TURBT for bladder invasion of PCa (mainly T4 cases), HoLEP, TUEB, HIFU, and subcapsular prostatectomy for BPH. Patients who received radical prostatectomy were not included.

### Treatments

Our institutional protocol was previously described [14–18]. In short, we basically prescribed 78 Gy (2 Gy per fraction) to the prostate and seminal vesicles for high-risk PCa, and 74–76 Gy (2 Gy per fraction) for low- and intermediate-risk PCa in conventionally fractionated IMRT, respectively, in which a total dose was reduced by 4 Gy in patients with a risk factor for rectal bleeding or urinary toxicities. Prophylactic pelvic irradiation was administered to patients with pelvic node metastasis [17] or some patients considered high-risk for nodal metastasis [19]. After February 2014, our institutions started moderately hypo-fractionated IMRT, in which 54 Gy in 15 fractions were prescribed for the prostate [16, 18]. In patients with a history of invasive interventions for the prostate prior to IMRT, the equivalent dose in 2-Gy fractions (EQD2) (α/β = 3) for normal tissues was reduced from 78 to 70–74 Gy. In hypo-fractionated IMRT of 54 Gy in 15 fractions, EQD2 was 71.3 Gy.

Our androgen deprivation therapy (ADT) method was described previously [14, 17, 20, 21]. In brief, 6-month neoadjuvant ADT with or without adjuvant ADT was performed according to the risk.

### Patient follow-up

Patients were followed every 1–3 months during the first 2 years, and every 3–6 months thereafter. Every examination included a checkup regarding GU and gastrointestinal (GI) toxicities, and involved prostate-specific antigen (PSA) and urinary tests. Patients were basically followed-up for at least 10 years after IMRT. Cystoscopy or urine cytology was basically performed for patients showing gross hematuria or continuous microhematuria on urine tests.

### Assessment of toxicity and oncological outcomes and statistical analysis

The primary objective of the current study was to evaluate the occurrence of late GU toxicity. Rates of late GI toxicity, overall survival (OS), and biochemical failure-free survival (BFFS) were also evaluated. Late toxicity was defined as that occurring more than 90 days after radiation completion, which was evaluated based on Common Terminology Criteria for Adverse Events (CTCAE) version 5.0. Incidental gross hematuria was recorded as grade 2. Any adverse event present prior to RT that did not worsen thereafter was not included as late toxicity. Hematuria or retention in patients with confirmed local recurrence was also excluded as late toxicity. Biochemical-failure was defined based on the Phoenix criteria (> 2.0 ng/mL elevation of PSA above nadir) [22].

Time zero for all events was set as the date of IMRT initiation. The cumulative incidence method was used to estimate rates of ≥ grade 2 and ≥ grade 3 GU and GI toxicities, accounting for death (or urinary diversion due to local recurrence for GU toxicities) without each event as a competing risk, and Kaplan–Meier estimation was used to evaluate OS and BFFS, respectively.

All statistical analyses were performed using EZR version 1.61 [23], a graphical user interface for R version 4.2.2 (The R Foundation for Statistical Computing, Vienna, Austria).

## Results

### Patient characteristics

A total of 35 patients met the criteria. Among them, three had an intervention history of TURBT for early-stage bladder cancer; the radiation field did not overlap with the area receiving TURBT in two, and the data of location data on TURBT were not available in one, respectively. Therefore, these three patients were excluded, and the remaining 32 were included in the analysis.

The median patient age was 75 (range: 54–83) years at the initiation of IMRT. More than two-thirds (*n* = 22) of patients were categorized into high-risk or higher groups according to the National Comprehensive Cancer Network risk classification (version 2, 2026) [24]. Of note, a quarter of the patients (*n* = 8) had clinical T4 disease with bladder neck invasion. At the start of radiotherapy, no patients had gross hematuria or a urinary catheter in use. The median interval between the invasive procedure involving the bladder or prostate and the subsequent radiation therapy for prostate cancer was 50.6 (interquartile range [IQR] 8.7–127.9) months.

Detailed characteristics of patients are summarized in Table 1.

**Table 1:** Patient and treatment characteristics

Patients and treatment characteristics		
Number of patients	32	
Age at IMRT(y), median (range)	75	(54–83)
Clinical T stage, n (%)		
T1-3	24	75.0%
T4	8	25.0%
NCCN risk classification (ver. 2.2026), n (%)		
Low or intermediate	10	31.3%
High	8	25.0%
Very high	7	21.9%
Node positive or metastatic	7	21.9%
With anticoagulant agents, n (%)	4	12.5%
Known diabetes metris, n (%)	4	12.5%
Subject to prior invasive interventions, n (%)		
Prostate cancer	13	40.6%
Benign prostatic hyperplasia	18	56.3%
Urinary stone impaction	1	3.1%
Type of prior invasive interventions, n (%)		
TURP	21	65.6%
HoLEP	5	15.6%
TURBT for bladder invasion of PCa	2	6.3%
HIFU	2	6.3%
TUEB	1	3.1%
Subcapsular prostatectomy	1	3.1%
IMRT doses/fractions for the whole prostate gland, n (%)		
78 Gy/39 fr	3	9.4%
74 Gy/37 fr	13	40.6%
72 Gy/36 fr	3	9.4%
70 Gy/35 fr	9	28.1%
54 Gy/15 fr	4	12.5%
IMRT with prophylactic pelvic irradiation, n (%)	6	18.8%

### Treatment characteristics

Conventional fractionated IMRT (2 Gy per fraction) was performed in 28 patients, and moderately hypo-fractionated IMRT was conducted in the remaining 4 patients. The median doses were 74 Gy in 37 fractions among patients treated with conventional fractionated IMRT, and 54 Gy in 15 fractions among patients treated with moderately hypo-fractionated IMRT. Regarding the 28 patients who received conventional fractionated IMRT, 11% (*n* = 3) were treated with 78 Gy in 39 fractions (un-reduced dose) to prioritize local control of the prostate, and 21% (*n* = 6) received prophylactic pelvic irradiation, whereas among the 4 patients who received moderately hypo-fractionated IMRT, 75% (*n* = 3) received simultaneous focal boost to 57 Gy for intra-prostatic dominant lesions identified on magnetic resonance imaging [18].

Details of patients and treatment characteristics are summarized in Table 1.

### Toxicities and oncological outcomes

**Fig. 1:**
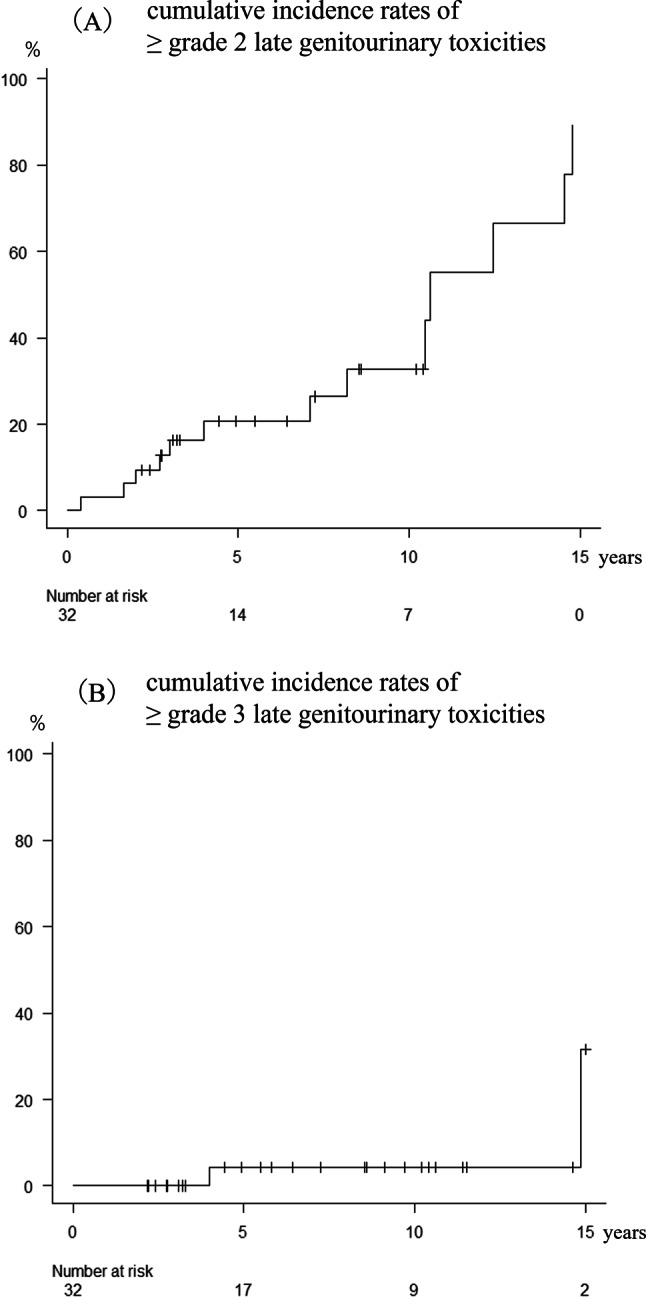
Cumulative incidence rates of ≥ grade 2 (**A**) or ≥ grade 3 (**B**) late genitourinary toxicities after intensity-modulated radiation therapy

**Fig. 2:**
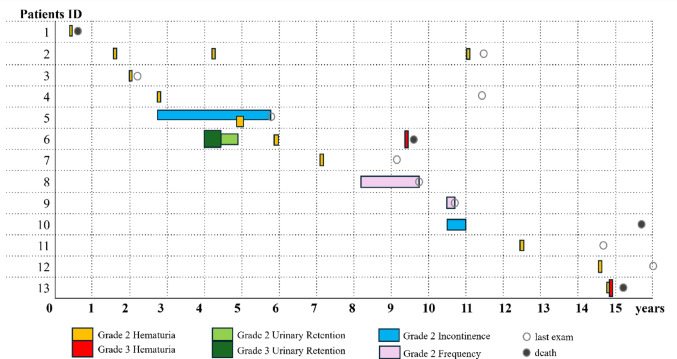
Patterns of occurrence of ≥ grade 2 late genitourinary toxicities after intensity-modulated radiation therapy

The median follow-up period was 77.3 (interquartile range [IQR] 38.1–122.9) months after IMRT. During follow-up, 40.6% (*n* = 13) developed grade 2 or 3 late GU toxicities, in which 77% (*n* = 10) experienced gross hematuria. Grade 3 late GU toxicities were observed in 6.3% (*n* = 2). No ≥ grade 4 late GU toxicity was noted. Although three grade 2 late GU toxicities, involving one case of incontinence and two cases of frequency, persisted throughout the observation period after occurrence, other late GU toxicities were transient and improved to grade 0 or 1 over time. Notably, 18.8% (*n* = 6) developed ≥ grade 2 late GU toxicity in a very late phase of more than 10 years after IMRT. Regarding hematuria, three patients developed grade 2 gross hematuria from 12 to 14 years, and one patient developed grade 3 gross hematuria at 15 years after IMRT. The cumulative incidence rates of ≥ grade 2 and ≥ grade 3 late GU toxicities were 20.7% (95% confidence interval [CI] 8.1–37.3) and 4.3% (95% CI 0.3–18.5) at 5 years, and 26.4% (95% CI 10.8–45.1) and 4.3% (95% CI 0.3–18.5) at 8 years, respectively (Fig. 1). Details of ≥ grade 2 late GU toxicities are shown in Fig. 2.

Concerning ≥ grade 2 late GI toxicities, one patient developed grade 3 rectal hemorrhage, which was controlled by endoscopic cautery without re-bleeding. Grade 4 or higher GI toxicities were not identified.

OS and BFFS rates were 89.5% (95% CI 70.4–96.5) and 76.0% (95% CI 55.8–87.9) at 5 years, and 89.5% (95% CI 70.4–96.5) and 60.7% (95% CI 37.9–77.3) at 8 years, respectively.

## Discussion

In this study, we evaluated late GU toxicities following prostate IMRT among patients with a history of invasive interventions for the prostate or bladder. To our best knowledge, the current study provides clinical data based on the longest reported follow-up period among IMRT research on such a population (median follow-up period: 77.3 months). As a result, the incidence of severe GU toxicities was relatively low. Specifically, the cumulative incidence rates of ≥ grade 2 and ≥ grade 3 late GU toxicities were 20.8 and 4.3% at 5 years, respectively, with no ≥ grade 4 late toxicity being observed. These results support the feasibility and safety of IMRT for such a population.

A history of prior invasive interventions for the prostate, such as TURP, HoLEP, or HIFU, has been reported as a risk for late GU toxicities following definitive RT for PCa [9, 10, 12]. According to the retrospective study by Devisetty et al., which evaluated GU toxicities following three-dimensional conformal radiotherapy or IMRT for PCa with a median dose of 72 Gy, a history of TURP was significantly correlated with a higher incidence of late ≥ grade 3 GU toxicities [10]. Specifically, regarding ≥ grade 3 GU toxicities, late GU toxicity was observed in 4% of patients without vs. 16% with previous TURP at 4 years (*p* = 0.0483). In our previous study, which investigated the occurrence of late GU toxicity following IMRT, the rate of ≥ grade 2 GU toxicities was 10% among patients without a history of prior invasive interventions for the prostate or bladder at a median follow-up of 104 months [8]. However, in the current study, ≥ grade 2 late GU toxicity was observed in 40.6% (*n* = 13 / 32) at a median follow-up of 77.3 months. Although not a direct comparison, late GU toxicities were frequent among patients with a history of such intervention. These results were consistent with previous reports.

More importantly, the incidence of severe late GU toxicities was relatively high among patients with a history of invasive interventions for the prostate or bladder [9–12, 25–32] (Table 2). According to the retrospective study by Singh et al., which evaluated late GU toxicities among 203 patients with history of prior TURP and received moderately or extremely hypofractionated IMRT, the cumulative rate of grade 3 late GU toxicities was 8.4%, and those toxicities mainly involved hematuria (6.4%) and urinary obstruction (3.4%), which were observed at a median of 29 months after IMRT and lasted for a median duration of 8 months [12]. Similarly, Takeda et al. reported that a history of HIFU was significantly correlated with a higher incidence of ≥ grade 3 late GU toxicities (*p* < 0.001), as well as a higher tendency of developing ≥ grade 3 late GU toxicities among patients with a history of TURP, although this was not significant (*p* = 0.153) [9]. Specifically, the 10-year grade 3 late GU toxicity-free survival rates were 50.0 and 92.1% among patients with and without prior HIFU, respectively, and 71.4 and 92.1% among patients with and without prior TURP, respectively. In the current study, grade 3 late GU toxicity was observed at 6.3% and ≥ grade 4 toxicities were not noted, which may be lower than previously reported considering our longer follow-up period. The chief symptom of grade 3 late GU toxicities was gross hematuria; they resolved to grade 0–1 with appropriate treatment. In our institution, we basically limited the dose to the prostate under EQD2 to 70 Gy, assuming an alpha/beta ratio of 3 Gy for patients with a history of invasive interventions for the prostate to minimize GU toxicities. Although speculative, this strategy may have contributed to the lower incidence of severe late GU toxicity. Therefore, it is considered important to maintain a balance between tumor control and the likelihood of late GU toxicity by modifying the treatment intensity.

**Table 2:** Studies on conventional fractionated and moderately hypo-fractionated prostate external-beam radiation therapy for patients with a history of invasive interventions for the prostate or bladder

Author	Year	Intervention prior to RT	Radiation technique	N (for late GU toxicity asssesment)	Dose / fraction [EQD2]	Median f/u	Late GU toxicities(Cumulative)
Devisetty et al.	2010	without TURP	3D-CRT (53%) IMRT(47%)	538	72 Gy (median)	50 m	G3 4% (4y)
		TURP	3D-CRT (69%) IMRT(31%)	71	70 Gy (median)	40 m	G2 45%, G3 16% (4y)
Takeda et al.	2021	without HIFU	IMRT	448	76–80 Gy/ 38–40 fr	83 m in 452 patients	G3 5.6%
		HIFU		4	76–80 Gy/ 38–40 fr		G3 50%
Maulik S et al.	2022	wtihout TURP	IMRT	82	66 Gy/ 20 fr [83.2 Gy]	70 m in 120 patients	G2 8%
		TURP		38	66 Gy/ 20 fr [83.2 Gy]		G2 42%
Singh et al.	2024	TURP	IMRT	114	68 Gy/ 25 fr [77.8 Gy]	51.5 m	G2 34%, G3 13%
Perennec et al.	2024	cryotherapy	IMRT	10	74–78 Gy(median 76 Gy)	60 m	G2 20%, G3 10%
Di Lalla V et al.	2022	HIFU	IMRT	9	76–78 Gy/38–39 fr, 66 Gy/ 22 fr [79.2 Gy]	46 m	G2 0%
The current study		TURP, HoLEP, TUEB, HIFU, adenectomy	IMRT	32	70–78 Gy/ 35–39 fr, 54 Gy/ 15 fr [71.3 Gy]	77 m	G2 20.8%, G3 4.3% (5y) G2 40.6%, G3 6.3%

In the current study, very late occurrence of GU toxicities following prostate IMRT was observed in 18.8% (*n* = 6) (Fig. 2), highlighting the importance of long-term follow-up for patients with a history of invasive interventions for the prostate or bladder. Of note, regarding hematuria, three patients developed grade 2 gross hematuria from 12 to 14 years, and one patient developed grade 3 gross hematuria at 15 years after IMRT, which improved to grade 0–1 after cauterization or conservative treatment. Considering its long latency and relatively high frequency of very late symptomatic GU toxicities among such a population, the typical follow-up period of EBRT for PCa would be insufficient. Therefore, our results suggest the importance of longer follow-up with close attention to symptomatic GU toxicities for such a population.

Our study had several limitations due to the retrospective nature of analysis. It consisted of a small cohort. In addition, previous interventions for our patients were not homogeneous, including TURP, TURBT, and HoLEP. Despite these limitations, our results were based on data following treatment with a relatively consistent IMRT method, as aforementioned. Considering the lack of prospective data regarding long-term GU toxicity after IMRT for patients with a history of invasive interventions for the prostate or bladder, our results serve as basic clinical data supporting the safety of IMRT for such a population.

In conclusion, IMRT for PCa was considered a safe and feasible treatment option for patients with a history of invasive interventions for the prostate or bladder, although the incidence of late GU toxicities was relatively high. Considering the existence of very late GU toxicities, sometimes more than 10 years after irradiation, long-term follow-up with close attention to the detection of GU toxicities is recommended for such a population.

## Data Availability

The data that support the findings of this study are available from the corresponding author (TM) on reasonable request.
